# PDMS/MWCNT-based tactile sensor array with coplanar electrodes for crosstalk suppression

**DOI:** 10.1038/micronano.2016.65

**Published:** 2016-12-19

**Authors:** Luxian Wang, Huiling Peng, Xiaolin Wang, Xiang Chen, Chunsheng Yang, Bin Yang, Jingquan Liu

**Affiliations:** 1 National Key Laboratory of Science and Technology on Micro/Nano Fabrication, Department of Micro/Nano Electronics, Shanghai Jiao Tong University, Shanghai 200240, China

**Keywords:** crosstalk suppression, PDMS/MWCNTs, tactile sensor array

## Abstract

The severe crosstalk effect is widely present in tactile sensor arrays with a sandwich structure. Here we present a novel design for a resistive tactile sensor array with a coplanar electrode layer and isolated sensing elements, which were made from polydimethylsiloxane (PDMS) doped with multiwalled carbon nanotubes (MWCNTs) for crosstalk suppression. To optimize its properties, both mechanical and electrical properties of PDMS/MWCNT-sensing materials with different PDMS/MWCNT ratios were investigated. The experimental results demonstrate that a 4 wt% of MWCNTs to PDMS is optimal for the sensing materials. In addition, the pressure-sensitive layer consists of three microstructured layers (two aspectant PDMS/MWCNT-based films and one top PDMS-based film) that are bonded together. Because of this three-layer microstructure design, our proposed tactile sensor array shows sensitivity up to −1.10 kPa^−1^, a response time of 29 ms and reliability in detecting tiny pressures.

## Introduction

Tactile sensors are used to detect subtle changes in the ambient environment and measure mechanical contacts with objects. Currently, tactile sensors with high sensitivity and flexibility exhibit good prospects in various applications such as artificial limbs, robot skin, touch screens, and wearable electronics. Normally, the operating mechanisms for tactile sensors can be divided into five categories: piezoelectric^1,2^, triboelectric^2^, optical^3,4^, capacitive^5–11^, and piezoresistive^12–30^. Because of the inherent stiffness of conventional piezoresistive materials, many piezoresistive tactile sensors are fabricated with the combination of flexible polymer materials such as polydimethylsiloxane (PDMS) and polyimide. However, their sensitivities are relatively low and require more deformation of microstructures under the same force^21^. To increase the sensitivity, Wang *et al*.^22^ fabricated a uniform and sensitive film with microstructures in a large area by molding with silk, which served as an electrical skin to monitor human physiological signals. Moreover, a layered tactile sensor design^23^ based on nanoscale mechanical interlocking between metal-coated nanofibers has been demonstrated to detect different types of loadings such as pressure, shear, and torsion. However, because the flexible polymer materials have poor dielectric properties, they will be difficult to use in the resistive tactile sensors. Recently, carbon-based materials such as graphene and carbon black have been used to improve the performance of tactile sensors. Zhu *et al.*^24^ developed a tactile sensor with excellent performance by depositing graphene films on the PDMS microstructures. Cheng *et al.*^25^ produced the tactile sensing element by dispensing a mixture of conductive PDMS prepolymer, nano-carbon black, nanosilver powder, and copper powder on the spiral electrodes, which can detect the twisting angle without damaging the sensor array. In addition to graphene and carbon black, another important carbon-based material is carbon nanotubes (CNTs), which have excellent mechanical and electrical properties^26^. Lipomi *et al.*^27^ produced a type of conductive, transparent, and stretchable nanotube film by directly spray-coating CNTs onto a PDMS substrate, and its resistance changed under different strains. Furthermore, CNT-doped PDMS has been used as an important material for pressure sensing. Han *et al.*^28^ and Pyo *et al.*^29^ proposed the development of piezoresistive pressure sensors based on printed PDMS/CNTs, which could detect three-dimensional forces. Combined with the pressure distribution measurement system, a printed multiwalled carbon nanotube (MWCNT)–PDMS composite pressure sensor proposed by Gerlach *et al.*^30^ is promising to avoid unhealthy rollover patterns by monitoring the plantar pressure.

In sensor design, the sandwich structure is commonly used in the tactile sensor array by depositing the electrode materials on both sides of the sensing materials along the co-axial direction of applied loading^31^. However, when the load is applied on the target sensing elements in the matrix, some effects on their adjacent elements induce the crosstalk. Therefore, the accuracy of the sensor array decreases accordingly.

This study presents a novel design for a PDMS/MWCNT-based resistive tactile sensor array to enhance the electrical isolation of each sensing element by separating them from each other in the same plane, which effectively solves the crosstalk problem. By optimizing the structure of the pressure-sensitive layer, the sensitivity, response time and detection limit of the sensor array are significantly enhanced. Furthermore, as the Au electrode layer is created on parylene as the polymer carrier, the sensor array has good flexibility and can be applied in various applications.

## Materials and methods

### Design of the sensor array

**Figure 1:**
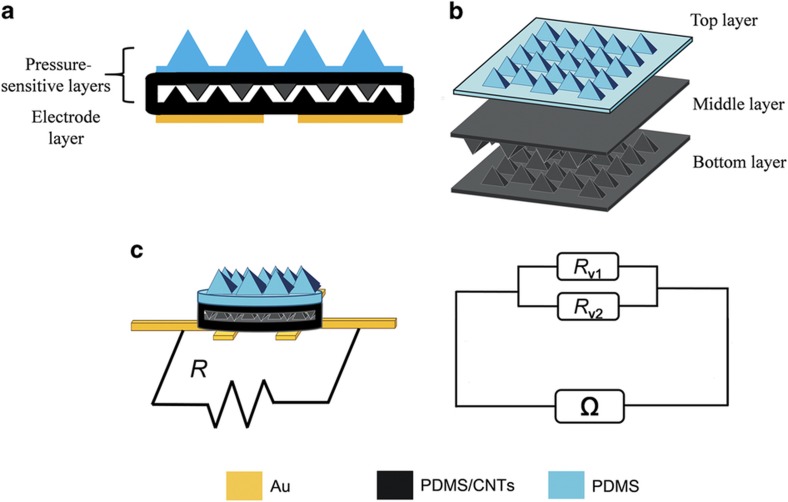
(**a**) Cross-section of the sensing element. (**b**) Magnified view of the pressure-sensitive layer. (**c**) Schematic diagram of the working principle of the sensor array.

The cross-section of a sensing element is shown in Figure 1a. Each sensing element consists of a pressure-sensitive layer and a coplanar Au electrode layer. As shown in Figure 1b, the pressure-sensitive layer is composed of three layers. The top layer is made of pure PDMS, and the middle and bottom layers are made of PDMS/MWCNTs. All three layers are designed to have a micropyramid structure to improve the sensitivity. The middle and bottom layers are assembled face to face, and the edges of these two layers are bonded together. The top layer is bonded on top of them. In particular, the PDMS-based top layer functions as a bump to better detect the mechanical contacts in the ambient environment and make the stress distribution become more concentrated.

Figure 1c illustrates the working principle of the sensor array. Because the edges of the bottom and middle layers are bonded together (Figure 1a), they can be considered two resistors connected in parallel. The output resistance *R* is defined as:

(1)R=(Rv1Rv2)/(Rv1+Rv2)

where *R*_v1_ and *R*_v2_ are the volume resistances of the bottom and middle layers, respectively. According to the percolation theory^32^ and general effective media theory^33,34^, the compressed nanocomposites increase the amount of conductive and percolating paths^35^. The volume resistance *R*_v_ is defined as^36^:

(2)Rv=q/p

where *q* is the piezoresistive coefficient, which is related to the type of material, and *p* is the pressure. First, the bump layer enables the stress to be more concentrative in the distribution, which increases the pressure on the top layer. Then, the micropyramids in the sensing layers enable the sensing element to have a greater degree of deformation, which leads to a larger interaction pressure and a more uneven pressure distribution. Hence, based on Equation (2), a larger difference in pressure corresponds to more obvious variations of *R*_v_. In addition, because both sides of the middle layer are free and the back side of the bottom layer is supported, the deformations of the middle and top layers are not identical. Thus, the resistance changes of *R*_1_ are not identical to that of *R*_2_, which makes the variations of *R* more obvious. The FEM results also prove this result (Supplementary Figure S1). However, a higher applied pressure to the sensor corresponds to a lower contact resistance. A compressive deformation of the pressure-sensitive layer enables the nanocomposites films in more contact with the Au electrodes, which results in more conductive pathways.

### Material preparation of the sensor array

PDMS (Sylgard 184 A, Dow Corning Company, Midland, MI, USA) doped with MWCNTs (XFM07, Nanjing XFNANO Materials Tech Co., Ltd, Nanjing, Jiangsu, China) was used as the sensing material. First, MWCNTs were dispersed in toluene and oscillated by ultrasonic waves for 2 h. Then, the PDMS prepolymer was added into the MWCNT/toluene solution and completely mixed by ultrasonic waves for 10 h. Afterward, the PDMS curing agent was added to the MWCNT-mixed prepolymer at the weight ratio of 1:10 for 30 min. Finally, the mixture was put in a vacuum chamber for 20 min to remove the bubbles for the following fabrication.

### Fabrication and assembly of the sensor array

**Figure 2:**
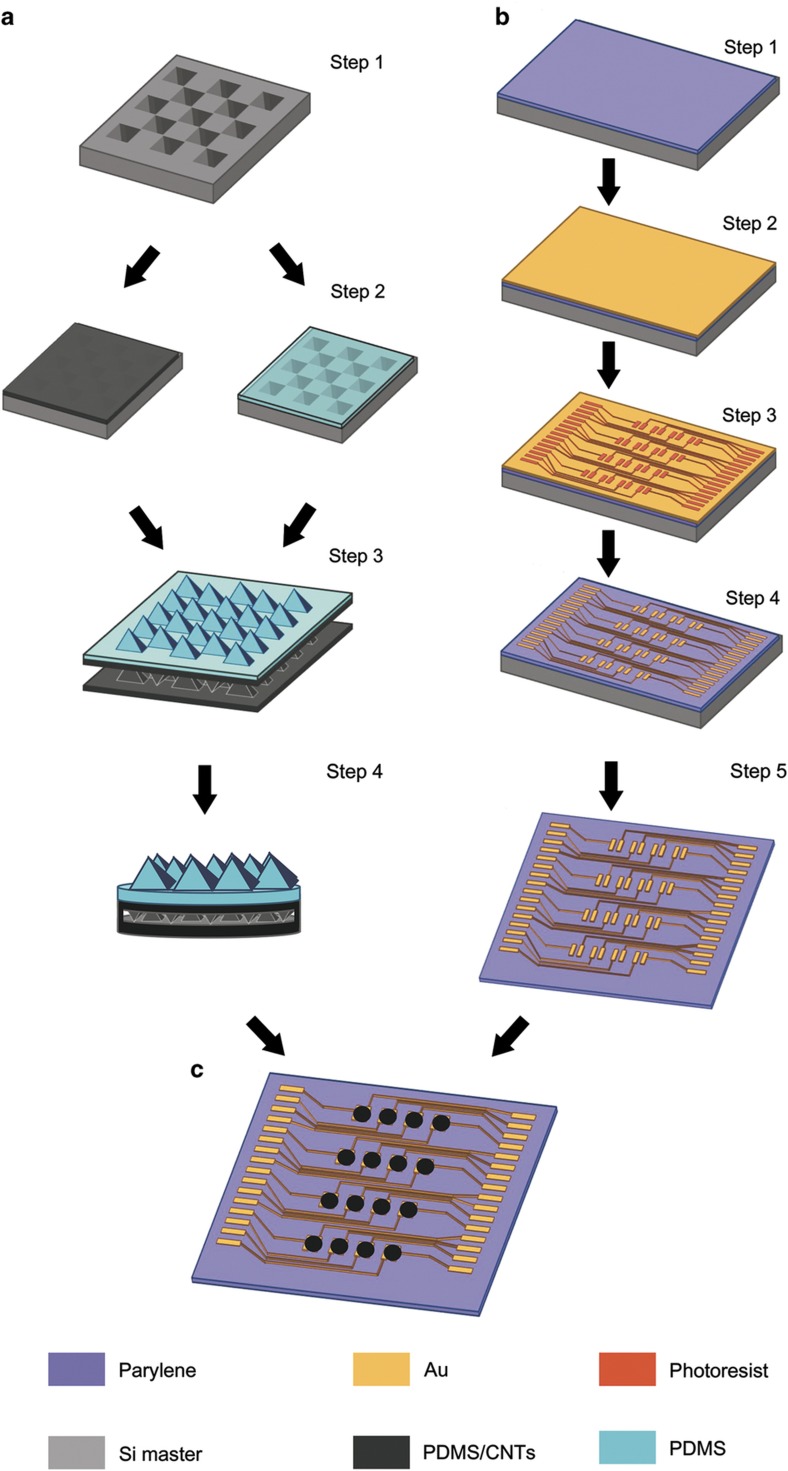
Fabrication process of the device. (**a**) Fabrication of the pressure-sensitive layer; (**b**) fabrication of the coplanar electrode layer; and (**c**) assembly of the sensor array.

The fabrication process of the tactile sensor array mainly includes the fabrication of a pressure-sensitive layer with micropyramids (Figure 2a) and a coplanar Au electrode layer (Figure 2b) and the assembly of the tactile sensor array (Figure 2c).

The fabrication process for the pressure-sensitive layer is shown in Figure 2a. The structure of the micropyramids was molded by an Si master, which can be fabricated by anisotropic wet etching through the SiO_2_ layer as the mask (Step 1 in Figure 2a). To decrease the adhesion between the Si mold and PDMS, a 5-μm-thick film of parylene was grown on the Si mold. Then, the toluene-diluted pure PDMS and MWCNT-doped PDMS were spin-coated on the mold (Step 2 in Figure 2a). Afterward, oxygen plasma was used to treat the surfaces of the MWCNT-doped PDMS layers, which can modify their surfaces to be hydrophilic. Subsequently, these two layers were immediately tightly assembled face to face at 60 °C for 15 min for permanent bonding. Because all areas except the 2-mm-diameter round element with micropyramids are flat, these two PDMS/MWCNT layers can be easily bonded together (Step 3 in Figure 2a). Finally, the round sensing elements with a diameter of 2 mm were punched with a seamless steel tube (Step 4 in Figure 2a). In addition, a smaller feature size of the microstructure corresponded to a higher sensitivity of the tactile sensor^24^. Therefore, the feature sizes of 50 and 200 μm were selected for the micropyramids in the nanocomposite layer and the bump layer, respectively.

**Figure 3:**
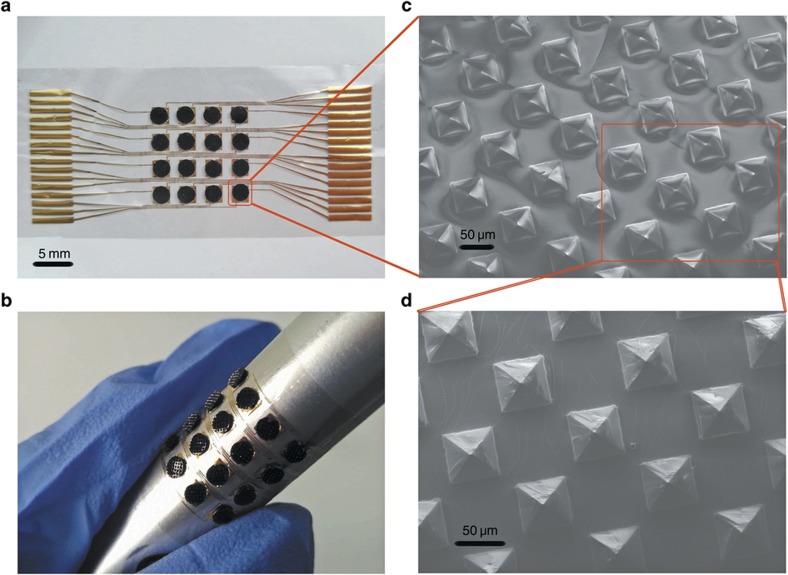
(**a**) The fabricated tactile sensor array. (**b**) The flexibility of the sensor array. (**c** and **d**) Scanning electron microscope images of 50-μm micropyramids incorporated in the sensing layer.

Figure 2b shows the fabrication process of the coplanar electrode layer. The electrode layer was fabricated on a highly flexible and stretchable thin parylene film, which was deposited on an Si wafer with a thickness of 5 μm (Step 1 in Figure 2b). Then, a 300 nm Au film was sputtered on the parylene film (Step 2 in Figure 2b) with subsequent photolithographic patterning (Step 3 in Figure 2b). Afterward, the electrode pattern was realized by the reactive ion etching method. The bottom electrode layer was obtained by removing the photoresist with acetone (Step 4 in Figure 2b) and peeled off for the assembly (Step 5 in Figure 2b). After fabricating the sensitive layers and electrode layer, the final step was to assemble these layers into the sensor array (Figure 2c). Figure 3a shows the prototype of the entire tactile sensor with excellent flexibility (Figure 3b). Figures 3c and d show the scanning electron microscope images of the 50-μm micropyramids in the top view with different magnifications.

## Results and Discussion

### Mechanical and electrical properties of PDMS/MWCNTs

**Figure 4:**
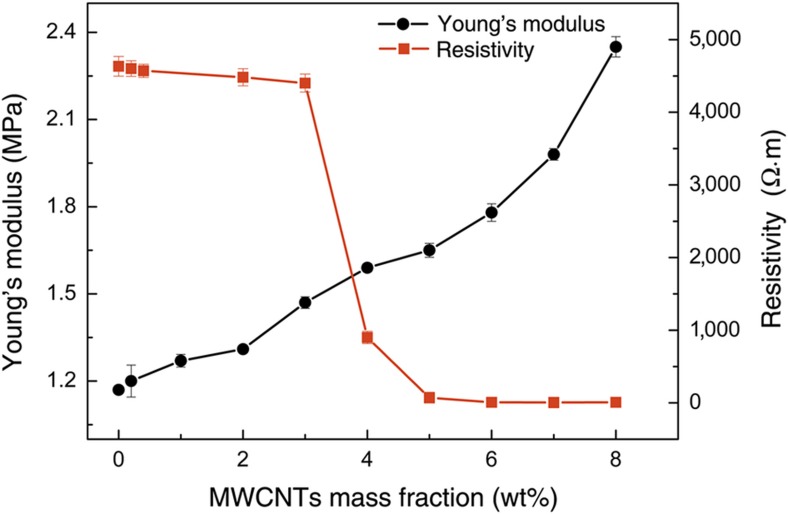
Mechanical and electrical properties of PDMS/MWCNT nanocomposites of different concentrations.

The concentration of MWCNTs significantly affects the mechanical and electrical properties of PDMS/MWCNTs, which can be characterized in terms of Young’s modulus and resistivity, respectively. The Young’s modulus was measured using a universal material testing machine (Z100, Zwick/Roell, Ulm, Germany). As shown in Figure 4, the Young’s modulus increases with the increase in MWCNT concentration in PDMS, which implies that the material with high MWCNT content has small deformation and low sensitivity under the same force. The main reason is that the CNTs with high stiffness (the axial Young’s modulus of CNTs is 1–5 TPa (Ref. 37)) result in the increase in Young’s modulus of nanocomposites.

For the resistivity changes of nanocomposites with different ratios, the testing results demonstrate that a higher MWCNT concentration corresponds to lower nanocomposite resistivity. Figure 4 shows a sharp decrease in resistivity at the mass fraction of 4 wt%. When the concentration is >5 wt%, the resistivity remains nearly constant. These results indicate that CNTs significantly improve the conductivity of nanocomposites. To balance the mechanical and electrical properties, PDMS/MWCNTs at a concentration of 4 wt% are finally selected as the optimal sensing materials.

### Characterizations of the sensor array

**Figure 5:**
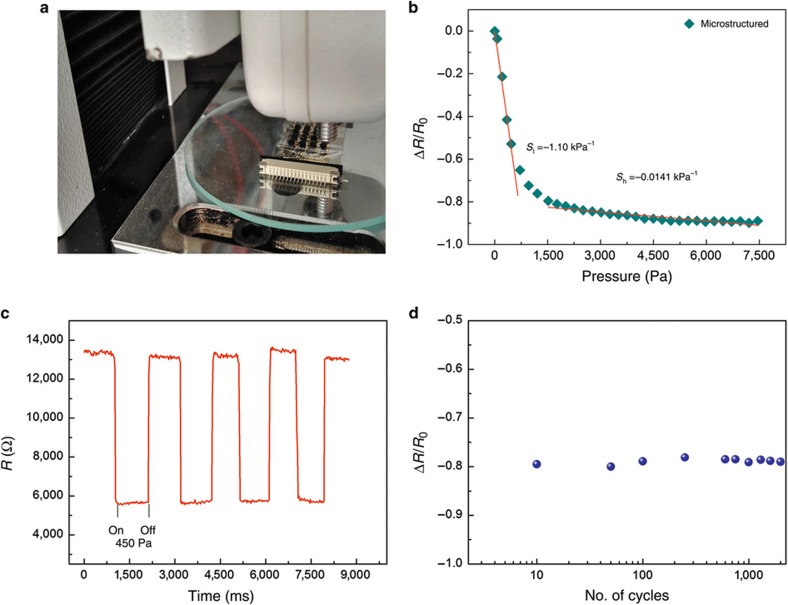
(**a**) Applying the pressure with a force gauge. (**b**) Pressure–response curves of the structures with microstructures. (**c**) Real-time resistance–time curve during the loading/unloading cycles (450 Pa). (**d**) Resistance changes after 2000 loading and unloading cycles with a pressure of 1 kPa.

**Figure 6:**
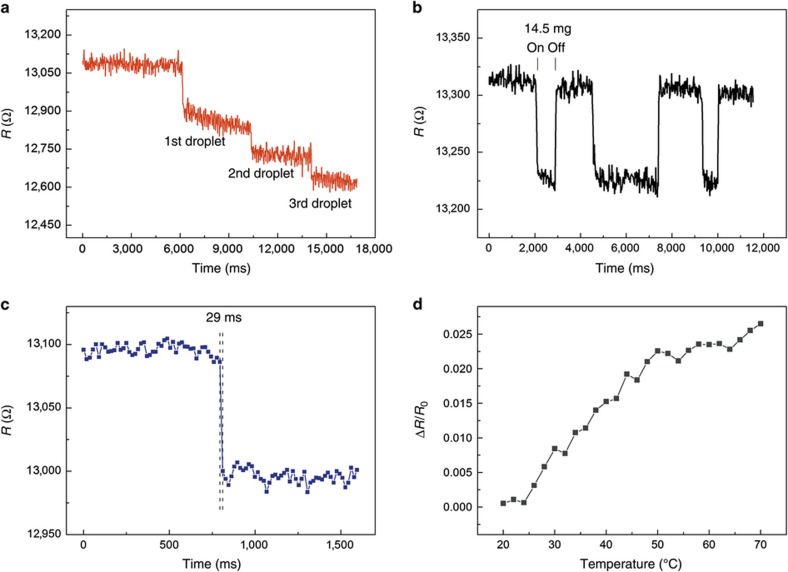
(**a**) Response of the sensor array to three continuous droplets. (**b**) The response of the sensor array to the loading/unloading of a small piece of non-woven fabric (14.5 mg). (**c**) Instant response time of the sensor array. (**d**) Temperature dependency of the sensor array.

To quantify the relationship between resistivity change and force, the applied pressure was gradually increased on one element of the sensor array using a force gauge (Figure 5a) and a motor-driven displacement platform. The resistance data were measured using an LCR meter (41100, Wayne Kerr, Shenzhen, China) under an alternative current voltage. For better measurement, the test frequency of the LCR meter was set at 500 kHz in the low-pressure regime (<1500 Pa) and at 1 kHz in the wide-pressure regime. The sensitivity *S* is defined as:

(3)S=∂(Δ(R−R0)/R0)/∂p

where *R*_0_ is the initial output resistance, and *R* is the output resistance when pressure *p* is exerted on the sensor element. As observed from the variation tendency of the resistance in Figure 5b, the resistance changes have a fast stage and a near-saturation stage. In the fast stage, the sensing layer markedly deformed in the small pressure range (0–800 Pa). The interaction pressure among the nanocomposite layers was significantly increased, which rapidly decreased *R*_v_. Thus, the output resistance *R* quickly decreased. At this stage, the sensitivity of the three-layer structure is −1.10 kPa^−1^. Because of the anisotropic structure of micropyramids^11^ in the near-saturation stage (>800 Pa), the interaction forces among the sensing layers and the deformation of the sensing layers slowly increased, which resulted in a relatively low sensitivity of −0.0141 kPa^−1^. Hence, this three-layer structure is proven to improve the sensitivity of the sensor array significantly. To study the dynamic of our sensor array, the device was repeatedly loaded/unloaded with identical pressure. To investigate the reliability of the sensor array in sensing low pressures, a pressure of 450 Pa was exerted on one sensing element. Figure 5c shows the resistance curve over time in four selected loading/unloading cycles, which demonstrates that the stable resistance changed without hysteresis in each cycle. The output resistance of the sensing element instantly returned to its initial value when the pressure was released. Negligible fluctuations in resistance changes are observed after 2000 cycles of loading and unloading with a pressure of 1 kPa (Figure 5d), which shows a good repeatability. Furthermore, to test the capability of detecting an extremely small force, we continuously dropped three droplets of water on one sensing element; the weight of each droplet was 40 mg. Figure 6a shows the continuous changes of the resistance of the sensing element. In addition, a tiny piece of non-woven fabric (14.5 mg) was put on one sensing element of the array. Figure 6b shows that the resistance of the sensor array significantly changed during the fabric loading/unloading process. Hence, the sensor array is similarly reliable in detecting the repeated loading and unloading of a tiny pressure. Moreover, a sharp resistance decrease with a response time of only 29 ms is observed, which proves the fast response of the sensor array, as shown in Figure 6c. In addition, the sensor array also shows a temperature dependency that the resistance increases with the increase in temperature from 20 to 70 °C. This result demonstrates that the sensor array is positively correlated with temperature (Figure 6d).

### Crosstalk study of the sensor array

Because all sensing elements in the coplanar electrode are separated in our sensor array, the electrical isolation between each sensing element can be significantly enhanced. To better study the crosstalk problem, we fabricated a sandwich-structured sensor array of similar sensitivity to our proposed sensor array.

**Figure 7:**
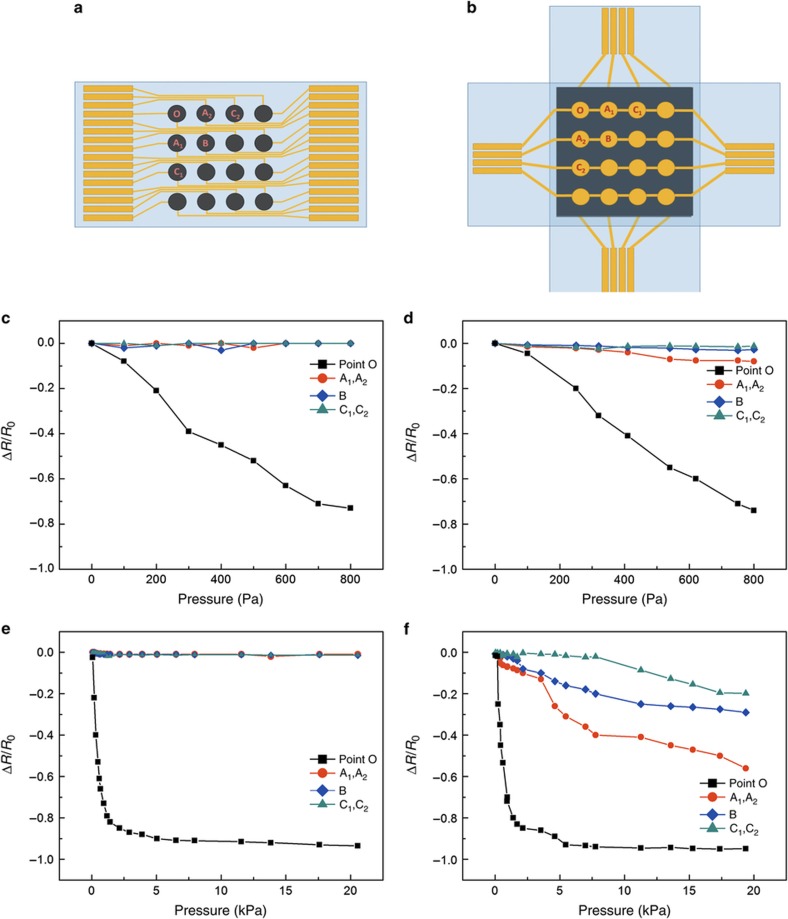
(**a**) Schematic diagram of the proposed sensor array. (**b**) Schematic diagram of the sandwich-structured sensor array. (**c** and **d**) Pressure–response curves of six designated elements in the low-pressure range. (**e** and **f**) Pressure–response curves of six designated elements in a wide-pressure range.

The schematic diagram of our 4×4 sensing array for the crosstalk study is shown in Figure 7a. The sensor array with the conventional sandwich structure (Supplementary Information) is shown in Figure 7b. Six sensing elements (O, A_1_, A_2_, B, C_1_, and C_2_) were selected as the testing points to investigate the crosstalk effects. Because of the increased distance from point O to the points of groups A, B, and C, it can better analyze the crosstalk effect in different regions of the sensor array. Because of the different sensitivity at different pressure ranges, the crosstalk effect in both low (<800 Pa)- and wide (0–21 kPa)-pressure ranges was investigated. A gradually increased force was applied at point O, and the resistance variations at these six points were simultaneously measured.

In a low-pressure range (Figure 7c), the resistance of point O of our proposed sensor array varied linearly, and the resistance variations of other selected points were close to zero. Although the sensitivity of the sensor array with the low-pressure range was high, the resistance values of points A_1_, A_2_, B, C_1_, and C_2_ remained stable, which indicates that the pressure at point O did not affect adjacent sensing elements. Similarly, the sandwich-structured sensor array shows a negative linear relation between resistivity change and pressure (Figure 7d). However, the resistance changes of points A_1_, A_2_, and B were more obvious than that of our designed sensor array.

In the wide-pressure range, as shown in Figure 7e, our proposed sensor array shows that the change in resistance at point O is consistent with the experimental result in Figure 5b. Although a large external pressure was applied, the resistances of adjacent elements (A_1_, A_2_, B, C_1_, and C_2_) remained close to zero. Thus, these results indicate that the electrical isolation among the sensing elements is ideal in both the low- and high-pressure ranges. In the sandwich-structured sensor array, as shown in Figure 7f, the change in resistance at point O tends to conform to the discovered experimental rules. Although the sensitivity in this large pressure range was relatively low, greater changes in resistance occurred at points A_1_, A_2_, B, C_1_, and C_2_. Therefore, the crosstalk effects at points A_1_, A_2_, B, C_1_, and C_2_ are inevitable and cannot be ignored in either pressure range. In addition, an important phenomenon is observed in this sandwich structure: the range of resistance variations depends on the distance from point O, which reflects an inverse relationship. By comparing the experiment results, we confirm that the effect of crosstalk is efficiently eliminated because of the superb electrical isolation among the sensing elements of our sensor array. Therefore, our sensor has excellent crosstalk suppression.

## Conclusions

In this study, we developed a novel tactile sensor array based on nanocomposites (PDMS/MWCNTs) with a micropyramid structure. To determine the optimal sensing materials, both mechanical and electrical properties of nanocomposites with different ratios of PDMS and MWCNT were investigated. By combining the isolated PDMS/MWCNT-based sensing elements in the coplanar electrode layers, the crosstalk problem was completely solved. Moreover, the pressure-sensitive layer design with pyramid microstructures can effectively improve the sensitivity of the sensor array. The device shows excellent static and dynamic properties, and the fabrication process is simple. Hence, our sensor array exhibits good prospects for applications in artificial limbs, robot skin, wearable electronics, and so on.
