# Supplementary material for: PDMS/MWCNT-based tactile sensor array with coplanar electrodes for crosstalk suppression
Source: Microsyst Nanoeng. 2016 Dec 19;2:16065. doi: 10.1038/micronano.2016.65 (PMC6444708; doi:10.1038/micronano.2016.65)
Supplement: Supplementary Information [file micronano201665-s1.pdf]

**Supplementary file**

# PDMS/MWCNT-based tactile sensor array with coplanar electrodes for crosstalk suppression

Luxian Wang, Huiling Peng, Xiaolin Wang, Xiang Chen, Chunsheng Yang, Bin Yang and Jingquan Liu

*Microsystems & Nanoengineering* (2016) **2,** 16065; doi:10.1038/micronano.2016.65; Published online: 19 December 2016

**Methods for alignment error reduction.** Thermal release tape (TRT) was used as a mask whose adhesive can be released after heating. We used laser to cut TRT out of the same layout and size of our designed sensing elements, and then pasted it on the electrode layer after alignment. Next, by applying a same amount of uncured PDMS/MWCNTs solution, each round sensing element was attached on its corresponding electrode pairs. Finally, after heating at 80 °C for 3 h, TRT could be peeled off and the sensing elements were fully cured.

**Fabrication of the sandwich-structured sensor array for comparison tests.** This sensor array for comparison tests was fabricated in a conventional way as shown in Figure 7b. Two independent large-area nancomposites films incorporated with micropyramids were assembled face-to-face. The bump layer with 200 μm micropyramids was bonded on top of the upper electrode layer. The size of every electrode pad is 2 mm in diameter which is with the same size as that of each sensing element in our proposed device.

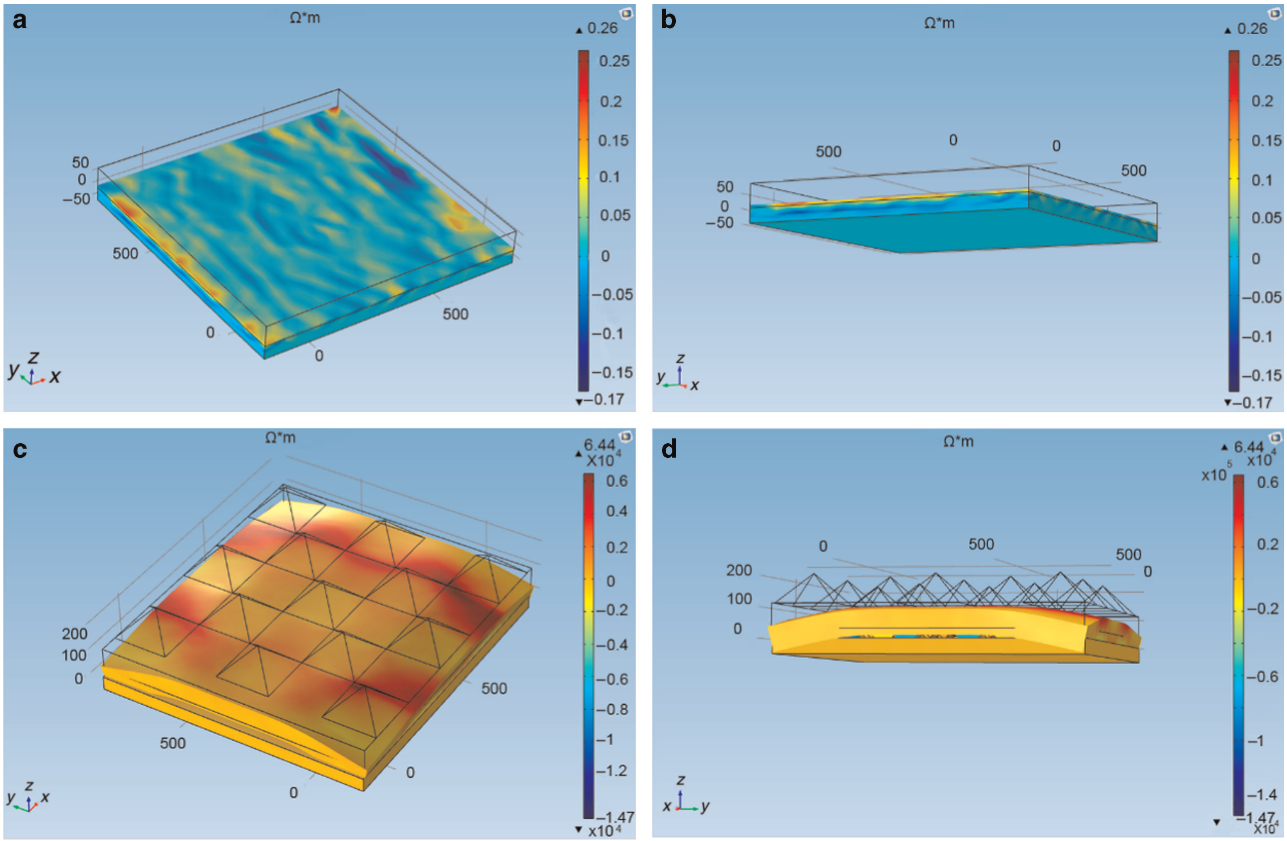


**Figure S1** FEM results of resistance changes of the structure with micropyramids from (**a**) top view and (**b**) side view. FEM results of resistance changes of the structure without micropyramids from (**c**) top view and (**d**) side view.

National Key Laboratory of Science and Technology on Micro/Nano Fabrication, Department of Micro/Nano Electronics, Shanghai Jiao Tong University, Shanghai 200240, China.
Correspondence: Jingquan Liu (jqliu@sjtu.edu.cn)
